# Allelopathic Molecular Mechanisms of the Two Main Allelochemicals in Sweet Potato

**DOI:** 10.3390/cimb46110706

**Published:** 2024-10-23

**Authors:** Ruiguo Shi, Guimei Jin, Shicai Shen, Gaofeng Xu, Fengping Zheng, David Roy Clements, Yunhai Yang, Shaosong Yang, Fanghao Wan, Fudou Zhang, Bo Liu

**Affiliations:** 1 College of Plant Health and Medicine, Qingdao Agricultural University, Qingdao 266109, China; shiruiguo.111@foxmail.com; 2 Key Laboratory of Prevention and Control of Biological Invasions, Ministry of Agriculture and Rural Affairs of China, Agricultural Environment and Resource Research Institute, Yunnan Academy of Agricultural Sciences, Kunming 650205, China; jgmbly2006@126.com (G.J.); shenshicai2011@aliyun.com (S.S.); xugaofeng1059@163.com (G.X.); zfping668@163.com (F.Z.); yangyunhainyx@163.com (Y.Y.); yshaos@163.com (S.Y.); 3 Key Laboratory of Green Prevention and Control of Agricultural Transboundary Pests of Yunnan Province, Agricultural Environment and Resource Research Institute, Yunnan Academy of Agricultural Sciences, Kunming 650205, China; 4 Yunnan Lancang-Mekong Agricultural Bio-Security International Science and Technology Cooperation Joint Research Center, Kunming 650502, China; 5 Department of Biology, Trinity Western University, Langley, BC V2Y 1Y1, Canada; clements@twu.ca; 6 Shenzhen Branch, Guangdong Laboratory of Lingnan Modern Agriculture, Genome Analysis Laboratory of the Ministry of Agriculture and Rural Affairs, Agricultural Genomics Institute at Shenzhen, Chinese Academy of Agricultural Sciences, Shenzhen 518000, China; wanfanghao@caas.cn

**Keywords:** sweet potato, palmitic acid, linoleic acid, allelopathic effects, transcriptomics, metabolomics

## Abstract

Sweet potato (*Ipomoea batatas* L.) is one of the most important global food crops. This crop exhibits excellent allelopathic potential against various weeds, but its allelopathic mechanism at the molecular level is unclear. Therefore, metabolomic and transcriptomic analyses were performed to explore the allelopathic effects, metabolic pathway, and associated genes for two major compounds with allelopathic activity, palmitic acid and linoleic acid. The sweet potato variety Ningshu 25 was employed in the current study. The results showed that palmitic acid and linoleic acid had strong allelopathic effects on seed germination, plant growth, antioxidant enzyme activity, and chlorophyll content of two weeds *Digitaria sanguinalis* and *Bidens pilosa*. The content of the two targeted metabolites was affected by different environmental conditions and was significantly increased under low temperature (15 °C). Five metabolic pathways involved in the two targeted metabolites of fatty acids were found: fatty acid biosynthesis, fatty acid elongation, fatty acid degradation, biosynthesis of cutin, suberine, and wax, and the linoleic acid metabolism pathway. The synthesis of palmitic acid is significantly enriched in the biosynthesis pathways of fatty acids, cutin, suberine, and wax, and the synthesis of linoleic acid is significantly enriched in the linoleic acid metabolism pathway. Under different environmental conditions, there were three key genes expressed—g4988, g11881, and g19673—located in the biosynthesis pathways of cutin, suberine, and wax; four key genes expressed—g31191, g60956, g49811, and g59542—located in the biosynthesis pathway of fatty acids; and six key expressed genes—g26575, g24787, g23517, g57649, g58562, and g4314—located in biosynthesis pathway of linoleic acid, respectively. Our study advances understanding of the molecular mechanisms behind allelopathic traits in sweet potato and provides a set of candidate genes for use in improving allelopathic potential in sweet potato germplasm resources.

## 1. Introduction

The new technology of intentionally using plant allelopathy for agricultural weeds has been generally considered one of the most promising bioengineering technologies for developing sustainable agriculture in the 21st century [1]. Field application of allelopathic crops has generated significant economic and ecological benefits. A good example is the use of allelopathic rice in the United States which has generated billions of dollars in benefits to agriculture [2]. Plant allelopathy is an ecological phenomenon that involves inhibitory or stimulatory direct or indirect effects of chemicals produced by plants impacting the growth, development and/or distribution of plants and/or microorganisms in the vicinity [3]. Serious problems caused by long-term and large-scale herbicide applications in agricultural production systems may be ameliorated by the use of crop allelopathy, including reductions in environmental pollution, human health issues, crop diversity losses, soil erosion, and herbicide-resistant weeds [4]. Finding ways of utilizing allelopathic crops through better understanding allelopathic mechanisms at ecological, physiological, and molecular levels could provide some of the solutions to reducing pesticide use and improved environmental protection.

The screening and cultivation of allelopathic germplasm resources from crop materials, as well as the exploration and utilization of allelopathic characteristic genes, have recently become a hot research topic [5,6,7]. The number of potentially allelopathic crops in proportion to the number of wild and cultivated crop resources to suppress weeds is relatively low. The allelopathic potential among crop varieties varies greatly [8]. This variation allows for breeding crop varieties with relatively high allelopathic potential, and such selective breeding has been utilized recently to develop systems to reduce herbicide use [5,6,7]. Crop allelopathy is produced via quantitative genetic traits under the control of multiple genes and does not have a direct relationship with the main agronomic characters [8]. The allelopathic traits of crops could be transferred to other commercial cultivars in order to produce new cultivars with weed-suppressing capability combined with high yield, as well as producing agronomic traits by using traditional cross and modern molecular breeding techniques [9]. Thus, genetic improvement of the allelopathic potential in crops is a promising breeding strategy for weed management for sustainable agricultural production.

Recent advances in molecular breeding technologies, such as genome-wide association studies (GWASs), DNA chips, next-generation sequencing (NGS), and meta-analysis, have facilitated advances in the genetic analysis of quantitative traits. Fine-scale linkage maps developed for quantitative trait locus (QTL) mapping is used to identify candidate’s genes and unravel complex trait architecture [10,11]. QTL mapping serves to inform fine gene mapping, gene cloning, and detection of relevant molecular markers for crop breeding [12,13]. Stable QTL and marker-assisted selection using molecular markers can be used effectively for the discovery of candidate genes to be used in transgenic or molecular introgression [14]. However, sequencing technology now facilitates high-throughput omics approaches, and multi-omics-combined analysis have been widely applied to explore the complex physiological mechanisms in plants, including plant species without genome maps [15,16]. Among the multi-omics technologies, integrated analyses of the transcriptome and metabolome in particular have been used to identify metabolic pathways, differently expressed genes, and their association in plants [17]. The association analysis of transcriptomes and metabolomes promises to make the breeding for allelopathic crop resources much more efficient.

Sweet potato (*Ipomoea batatas* L.) is an herbaceous vine native to Central America that can be annual or perennial and serves as an important cash and food crop in many regions around the world. Despite its value as a food crop, the sweet potato exhibits a broad tolerance range in terms of ecological, climate, and environmental conditions. Sweet potato has been shown to be highly competitive with various weeds owing to its prolific asexual reproduction, rapid growth, and extensive canopy coverage [18]. Morphological and physiological inhibition of a variety of invasive alien weed species is observed when grown with sweet potato [18,19,20,21]. Moreover, chemical interference has increasingly been implicated in enhancing the competitive effects of sweet potato on various plants [22,23,24,25,26]. In our previous study, some chemical components of petroleum ether extract of sweet potato were characterized and identified [26]. However, the genetic basis of these allelopathic traits in sweet potato has not previously been determined.

Based on chemical component identification of sweet potato [26], the principle objectives of the current study were to examine the allelopathic effects, metabolic pathways, and genes associated with palmitic acid and linoleic acid, identified as the two main components of sweet potato allelopathy, in controlled laboratory conditions by using a combination of transcriptome and metabolome analyses. Through this work, we aim to identify candidate genes associated with allelopathic traits and helping in marker-assisted selection for the improvement of allelopathic characteristics in sweet potato.

## 2. Materials and Methods

### 2.1. Study Species

Based on our previous allelopathic assessment of sweet potato germplasm resources widely grown in Yunnan Province, China [27], the variety Ningshu 25 with high allelopathic effects was selected. This variety has been collected and grown in the greenhouses at the Agricultural Environment and Resource Research Institute, Yunnan Academy of Agricultural Sciences since 2012. In September–October 2022, seeds from local populations of two noxious weeds *Digitaria sanguinalis* L. and *Bidens pilosa* L. were dried at room temperature and then stored at −4 °C. *Digitaria sanguinalis (*monocotyledonous plant in the Poaceae) and *B*. *pilosa* (dicotyledonous plant in the Asteraceae) are common weeds in most temperate, subtropical, and tropical regions of the world and are widely found in various habitats including greenhouses, nurseries, arable land, gardens, roadsides, and wasteland areas.

### 2.2. Collection and Extraction of Materials

The experiments were conducted on Ningshu 25 under different environmental conditions (CK, 25% shading rate covered by a black nylon shade netting, 15 °C, and 25 °C) in controlled greenhouses. On 10 June 2023, uniform three-node segments were taken from the central stem portions of relatively young Ningshu 25 plants and then transplanted in such a way as to produce a constant planting density of 9 plants m^−2^ (0.50 m × 0.50 m space) in 9 m^2^ plots (3 m × 3 m). All plots were arranged in a complete randomized design with each environmental condition replicated 4 times. All plants were distributed evenly within the plot and weeded by hand. No synthetic fertilizers were used during the experiment.

On 10 August, about 400 g of fresh whole plants of the Ningshu 25 was collected in each plot. Harvested plants were washed several times with distilled water to eliminate soil particles and then cut into pieces (approximately 1–2 mm) to extract components. Samples were individually placed in conical flasks (4 L), and distilled water was added. Subsequently, the filtered aqueous extract was partitioned with the solvent petroleum ether and then was dried using a rotary evaporator at 35 °C and stored in the dark at −4 °C.

For transcriptome sequencing, leaves weighing 10–15 g of Ningshu 25 were sampled from each plot and frozen in liquid nitrogen immediately after harvesting.

### 2.3. Assessment of Allelopathic Effects

The bioassay experiments utilizing palmitic acid and linoleic acid (the two major sweet potato compounds showing potential allelopathic activity) on two noxious weeds *D. sanguinalis* and *B. pilosa* were carried out in the laboratory. Five aqueous concentrations (0.125, 0.25, 0.5, 1.0, and 2.0 mg·mL^−1^) and a control (distilled water) (CK) with four replications were used to test each compound. For each treatment, 30 sterile seeds of each of the two weed species were distributed uniformly in 9 cm Petri dishes, with 5 mL of either extract or distilled water (control) added. Samples were collected to determine the germination rate, shoot height, root length, and fresh biomass within 7 days. Moreover, the physiological effects of palmitic acid and linoleic acid on two bioassay weed seedlings were evaluated by measuring levels of antioxidant enzymes superoxide dismutase (SOD), catalase (CAT), peroxidase (POD), chlorophyll-a, and chlorophyll-b.

### 2.4. HPLC-MS Analysis

High-purity standards of palmitic acid and linoleic acid were obtained by purchasing them from Aladdin Biochemical Technology Co., Ltd., Shanghai, China. Metabolic profiling of the extracts for targeted secondary metabolites of 16 samples was performed using an Agilent 1290 Infinity UHPLC-MS system (Agilent Technologies, Santa Clara, CA, USA). The UHPLC-MS analysis procedures of two targeted secondary metabolites of 16 samples under different environmental conditions conformed to the procedures followed by Latif et al. [28]. The quality control samples were analyzed using a blank for comparison and a standard mixture of two targeted metabolites (palmitic acid and linoleic acid) at a 1.5 mg·mL^−1^ concentration.

### 2.5. Transcriptome Sequencing and Analysis

The transcriptome sequencing was carried out by Novogene (Wuhan, China). Standard extraction methods were used to extract and isolate total RNA from each frozen sample. The integrity of the RNA samples was assessed using Agilent 2100 bioanalyzer (Agilent Technologies, Santa Clara, CA, USA). The RNA-seq was conducted using Illumina Novaseq (San Diego, CA, USA, PE150). The RNA-seq assembly and analysis were performed by Wuhan Benagen Technology Company (Wuhan, China). Quality control for the data analysis involved filtering the raw sequence data by removing low quality reads and adaptors in order to transform raw sequences into clean reads. Clean reads were checked for the sequencing error rate and distribution of GC content. The clean reads were compared to the reference genome using HISAT2 2.2.1 software (Johns Hopkins University, Baltimore, MD, USA) to produce localization information of the reads on the reference genome [29]. StringTie 2.1.4 software (Johns Hopkins University, Baltimore, MD, USA) was used to conduct new transcript assembly and calculate the level of gene expression for FPKM. DESeq2 4.1.1 software (Max Planck Institute for Molecular Genetics, Berlin, Germany) was used to perform differential analysis with genes considered differentially expressed when they had FDR < 0.05 and |log2Fold Change| ≥ 1 were subjected to KEGG analysis.

### 2.6. Integrated Analysis of Metabolomics and Transcriptomics

The metabolites and differentially expressed genes were analyzed in combination with KEGG to investigate the relationships between genes and metabolites. Plotting the heat map pathway allowed us to discern which metabolites and corresponding differential genes were most significant. WGCN analysis was used to look at the correlation between genes and metabolites.

### 2.7. Statistical Analysis

We calculated the allelopathic response index of aqueous solutions from *D. sanguinalis* and *B. pilosa* (RI: when T ≥ C, RI = 1-C/T; when T < C, RI = T/C-1; C is the control value and T is the treatment value), demonstrated by Williamson and Richardson to be the most effective index [30]. Statistical differences among the data were evaluated with one-way analysis of variance (ANOVA) and Tukey’s multiple range tests at a 5% level of significance. These analyses were conducted using IBM SPSS 23.0 software (Armonk, New York, NY, USA).

## 3. Results

### 3.1. Seed Germination and Seedling Growth

The results showed that both seed germination and seedling growth of t *D. sanguinalis* and *B. pilosa* were significantly affected by the solutions of palmitic acid and linoleic acid (Table 1 and Table 2). All measured allelopathic indices of *D. sanguinalis* and *B. pilosa* were significantly lower than 0 at concentrations of 0.125–2.00 mg·mL^−1^ and were significantly reduced with increasing concentration. Among the components tested for allelopathic response, it was found that biomass of two weeds was the most strongly suppressed, followed by root length and shoot length, and finally, germination rate was the least affected. The inhibition rates of linoleic acid on seed germination and plant growth were higher than those of palmitic acid (Table 1 and Table 2).

### 3.2. Physiological Effects

The enzyme and chlorophyll characteristics of the seedlings of the two weed species varied significantly (*p* < 0.05) among palmitic acid and linoleic treatments (Table 3 and Table 4). The SOD and POD activities for *D. sanguinalis* seedlings were lower than those of the CK, and markedly decreased with increasing concentration of the two sweet potato components. The CAT activity of *D. sanguinalis* seedlings and activities of SOD, CAT, and POD of *B. pilosa* seedlings were significantly increased with increasing concentration of the two sweet potato components. The chlorophyll-a and chlorophyll-b contents of *D. sanguinalis* and *B. pilosa* seedlings were markedly decreased with increasing concentrations of palmitic acid and linoleic acid (Table 3 and Table 4). Similarly, the inhibition rates of linoleic acid on enzyme and chlorophyll characteristics of two weeds were higher than those of palmitic acid (Table 3 and Table 4).

### 3.3. Metabolomics Analysis of Two Targeted Metabolites

**Figure 1:**
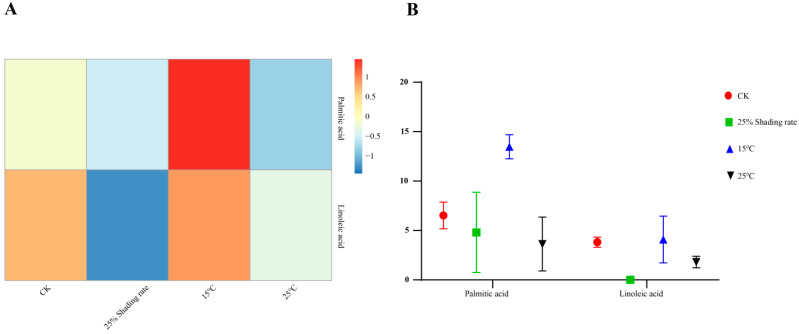
Cluster heat map (**A**) and bar chart (**B**) of differential metabolites clustering in Ningshu 25 under different environmental conditions.

The contents of the two main components, palmitic acid and linoleic acid, in Ningshu 25 were different under different environmental conditions (Table 5 and Figure 1). The content of palmitic acid under 15 °C treatment was significantly higher than the control. The contents of palmitic acid under 15 ^°^C, the control, 25% shading rate, and 25 °C treatments were occupied by 13.47%, 6.52%, 4.81%, and 3.63% of total component contents, respectively. Under 25 °C treatment, the content of linoleic acid was significantly higher than that of the control. The contents of linoleic acid under 15 °C, the control, 25 °C, and 25% shading rate treatments, were occupied by 4.09%, 3.82%, 1.81%, and 1.05% of total component contents, respectively.

### 3.4. Differential Expression Gene Screening of the Two Targeted Metabolites

**Figure 2:**
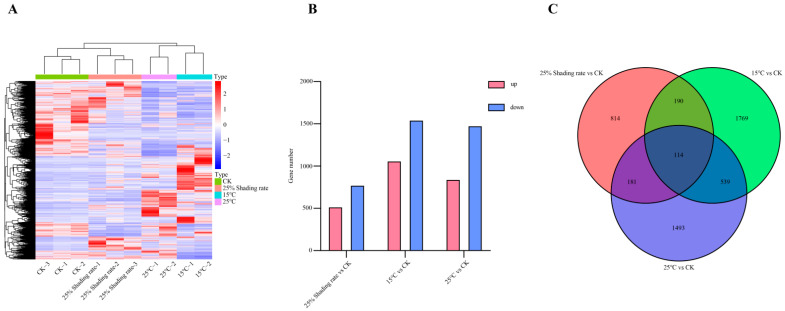
Differential gene analysis ((**A**) = heatmap of DEGs, (**B**) = bar chart of the number of upregulated and downregulated genes and (**C**) = venn diagram of DEGs) of Ningshu 25 under different environmental conditions.

The screening conditions for differential genes in this study were |log2Fold Change| ≥ 1 with FDR < 0.05. A total of 5100 different genes were screened out in the 15 °C, 25 °C, and 25% shading rate groups. In the group with 25% shading rate vs. CK, 1299 differential genes were screened out, of which 521 genes were upregulated and 778 were downregulated. A total of 2612 different genes were screened out in the 15 °C vs. CK group, of which 1064 genes were upregulated and 1548 were downregulated. In the 15 °C vs. CK group, a total of 2612 different genes were screened out of which 1064 genes were upregulated and 1548 were downregulated. In the group of 25 °C vs. CK, 2327 differential genes were screened out, of which 846 were upregulated and 1481 were downregulated (Figure 2).

### 3.5. Integrated Metabolomic and Transcriptomic Analysis

**Figure 3:**
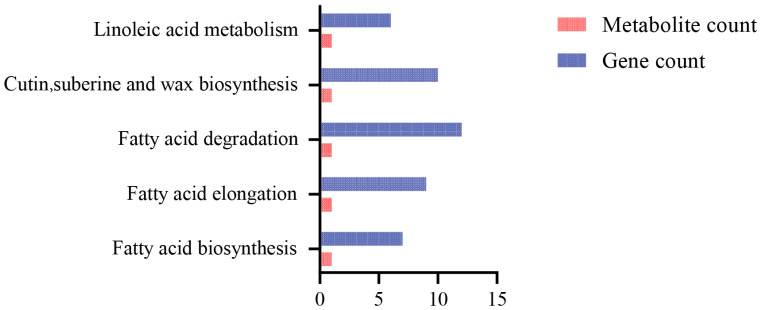
KEGG enrichment pathway analysis of metabolites and differential genes in Ningshu 25 under different environmental conditions.

In the KEGG enrichment analysis (Figure 3), it was found that palmitic acid and linoleic acid are involved in five metabolic pathways related to fatty acids, including fatty acid biosynthesis, fatty acid elongation, fatty acid degradation, biosynthesis of cutin, suberine and wax, and the linoleic acid metabolism pathway. The synthesis of palmitic acid was significantly enriched in the biosynthesis pathways of fatty acids, cutin, suberine, and wax, and the synthesis of linoleic acid was significantly enriched in the linoleic acid metabolism pathway. Comparing the metabolic pathways of differential expression genes, there were 7, 9, 12, 10, and 6 differentially expressed genes significantly enriched in the fatty acid biosynthesis pathway, fatty acid elongation pathway, fatty acid degradation pathway, biosynthesis pathway of cutin, suberine, and wax, and linoleic acid metabolism pathway, respectively (Figure 3).

**Figure 4:**
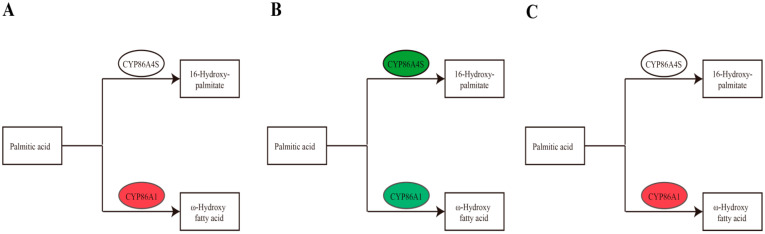
Biosynthetic pathways of cutin, suberine, and wax in Ningshu 25 under different environmental conditions ((**A**) = under 25% shading, (**B**) = under 15 °C and (**C**) = under 25 °C).

In the KEGG enrichment analysis for the cutin, suberine, and wax biosynthesis pathways, in the group with 25% shading rate vs. CK, the expression levels of the key genes g4988 of long-chain fatty acid omega-monooxygenase enzyme and g11881 of fatty acid omega hydroxylase enzyme were significantly upregulated (Table 6 and Figure 4A). Long-chain fatty acid omega-monooxygenase enzyme and fatty acid omega hydroxylase enzyme belong to an aliphatic hydroxylase identified as a key enzyme in the biosynthesis of arbutin in *Arabidopsis* aliphatic roots. In the group of 15 °C vs. CK, the expression level of the key gene g19673 of fatty acid omega-hydroxylas enzyme was obviously downregulated (Table 6 and Figure 4B), and in the group of 25 °C vs. CK, the expression level of the key gene g4988 of long-chain fatty acid omega-monooxygenase enzyme was significantly upregulated (Table 6 and Figure 4C).

**Figure 5:**
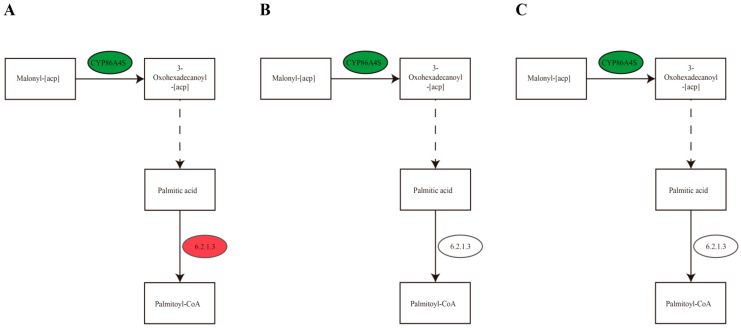
Biosynthesis pathway of fatty acid biosynthesis pathways in Ningshu 25 under different environmental conditions ((**A**) = under 25% shading, (**B**) = under 15 °C and (**C**) = under 25 °C).

In the KEGG enrichment analysis of the biosynthesis pathway of fatty acids, in the group with 25% shading rate vs. CK, the expression levels of the key genes g31191 and g60956 of 3-oxoacyl-[acyl-carrier-protein] synthase II enzyme were significantly downregulated, and the expression level of the key gene g49811 of long-chain acyl-CoA synthetase enzyme was significantly upregulated (Table 7 and Figure 5A). The 3-oxoacyl-[acyl-carrier-protein] synthase II enzyme is similar to dehydrogenase or condensation enzyme that is typically involved in the synthesis of fatty acids and similar molecules. The long-chain acyl-CoA synthetase enzyme in fatty acid degradation is controlled by the transcription regulatory factor FadR. In the groups of 15 °C vs. CK and 25 °C vs. CK, the expression levels of the key genes g60956, g31191, g59542, and g60956 of 3-oxoacyl-[acyl-carrier-protein] synthase II enzyme were clearly downregulated (Table 7 and Figure 5B,C).

**Figure 6:**
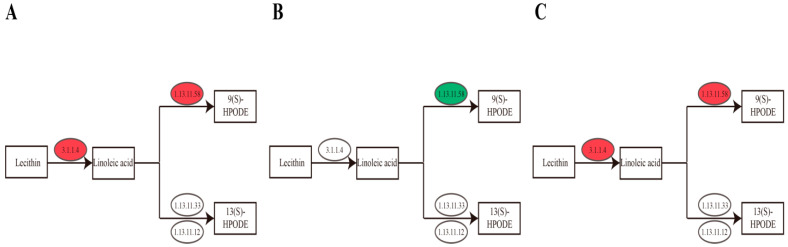
Biosynthesis pathways of linoleic acid in Ningshu 25 under different environmental conditions ((**A**) = under 25% shading, (**B**) = under 15 °C and (**C**) = under 25 °C).

In the KEGG enrichment analysis for the biosynthesis pathway of linoleic acid, in the group with 25% shading rate vs. CK, the expression levels of the key gene g26575 of secretory phospholipase A2 enzyme, g24787 of TAG lipase enzyme and g23517 of linoleate 9S-lipoxygenase enzyme were significantly upregulated (Table 8 and Figure 6A). The secretory phospholipase A2 enzyme catalyzes the hydrolysis of sn-2 ester bonds in various phospholipids, TAG lipase hydrolyzes triacylglycerol (TAG) to produce free fatty acids and glycerol, and linoleate 9S-lipoxygenase is a type of lipoxygenase that catalyzes the oxidation of unsaturated fatty acids to form hydrogen peroxide derivatives [31]. In the group of 15 °C vs. CK, the expression level of the key gene g23517 of linoleate 9S-lipoxygenase enzyme was markedly downregulated (Table 8 and Figure 6B). In the group of 25 °C vs. CK, the expression levels of the key genes g5764 and g58562 of TAG lipase enzyme were clearly upregulated, and the expression level of the key gene g4314 of lipoxygenase enzyme was evidently downregulated (Table 8 and Figure 6C). Lipoxygenase is a type of lipoxygenase that catalyzes the oxygenation of linoleic acid producing hydrogen peroxide in the process.

## 4. Discussion

Palmitic acid and linoleic acid are widely present in crops as potential allelochemicals depending on concentration and other factors [26,32,33,34] and may suppress plant growth, modify SOD and POD activity and the proline content of many plant species [35,36,37], and change the soil rhizosphere’s microbial and nutrient composition [38]. Our previous study showed that palmitic acid and linoleic acid are the major components exhibiting potential allelopathic activity in sweet potato [26]. The current study demonstrated that palmitic acid and linoleic acid strongly inhibited two weed species *D. sanguinalis* and *B. pilosa*, further confirming that these two components are the main allelochemicals of sweet potato. Therefore, it is of value to understand the allelopathic metabolic pathways and associated genes of palmitic acid and linoleic acid in sweet potato.

The level of allelopathic activity primarily depends on the target species, extract concentrations, and compound types [25,39]. Seed germination and morphological index are the most common measured indicators for plant allelopathic research. Seed germination or seedling growth of the target species is usually inhibited at high solution concentrations but may be promoted at low solution concentrations [40]. The population density and importance values of *D. sanguinalis* and *B*. *pilosa* significantly declined as sweet potato cover increased in sweet potato fields [41]. The current study found that germination, plant growth, and biomass of *D. sanguinalis* and *B*. *pilosa* were markedly inhibited by palmitic acid and linoleic acid, and the effects were concentration-dependent. Comparing allelopathic effects of the two compounds on the two weed species, we found that linoleic acid exhibited greater inhibition than palmitic acid. Biomass was the most inhibited, followed by root length and shoot length, with germination rate showing the least inhibition.

The physiological effects of allelochemicals on seed germination and plant growth are also important measured indicators for allelopathic potential. Our study found that the CAT content of *D. sanguinalis* and the SOD, CAT, and POD contents of *B. pilosa* were significantly increased by palmitic acid and linoleic acid, whereas SOD and POD contents of *D. sanguinalis* decreased. Similarly, the SOD, CAT, and POD activities of *B. pilosa* were increased with increase in the aqueous extract concentration of invasive alien plant *Tithonia diversifoli* [42]. Antioxidant enzymes are among the important reactive oxygen detoxifier systems in plant cells; thus, an increase in the activity of antioxidant enzymes activity could represent a key defense strategy against oxidative stress [43]. A major reason for the importance of oxidative stress is its impact on plant growth through the inhibition of photosynthesis and respiration. Higher rates of photosynthesis connected to higher chlorophyll content can lead to increased growth rates, biomass accumulation, and overall production. Conversely, increased growth and accumulation of biomass results from higher rates of photosynthesis associated with higher chlorophyll levels [19]. In the present study, we found that both chlorophyll-a and chlorophyll-b contents of *D. sanguinalis* and *B. pilosa* were greatly reduced with increasing concentration of palmitic acid and linoleic acid. Therefore, it is clear that these two compounds modify the activity of the antioxidant enzymes SOD, POD, and CAT and reduce chlorophyll content in *D. sanguinalis* and *B. pilosa*, thus affecting the germination rate and seedling growth of two weeds.

Crop allelopathy is a complicated chemical ecological phenomenon that may be regulated by various environmental factors [44,45]. Changes in environmental conditions can affect the synthesis, content, and types of allelochemicals in crops resulting in varying allelopathic effects [46]. Crop allelopathic activity could often be enhanced under stress environment such as lower temperature, weak sunlight, and lack of nutrients [47]. Temperature and light are the principal environmental factors affecting allelopathic potential at different crop growth stages [48,49]. Crop allelopathy can be increased or decreased by temperature and light conditions; for example, allelopathic effects tend to be weakest under low temperature [50,51]. Thus, the understanding of how environmental conditions regulate allelopathic effects has been regarded as a key means for improving allelopathic efficacy in agricultural production. The current study found that palmitic acid and linoleic acid contents of the Ningshu 25 sweet potato variety varied under different environmental conditions. In comparison to the control, the percentage of the two components was increased under 15 °C and decreased under 25% shading rate and 25 °C treatments. The likely explanation is that a 25% shading rate and 25 °C are optimal growth conditions for sweet potato.

Plant allelopathy is a quantitative trait determined by multiple genes and mediated by environmental factors [9]. In order to explore the metabolic pathways and associated genes of crops with allelopathic traits, the combination of transcriptome and metabolome analysis has been widely used under different environmental conditions [52]. Metabolomics, as a method for detecting the metabolome within plants, reveals the accumulation patterns of metabolites across different species, various tissues within the same species, and the same tissue under different stress conditions. This provides a reference for identifying plant-specific accumulated metabolites and marker metabolites in response to stress [53]. The integration of metabolomics and transcriptomics allows for the organic combination of life processes and outcomes and is taking on an increasingly important role in the identification of functional genes and metabolic pathways [54]. This current study employed metabolomics and transcriptomics to conduct a co-expression analysis of differential genes and metabolites, identifying the metabolic pathways and important genes involved in the synthesis of palmitic acid and linoleic acid in sweet potato.

We found that under the 25% shading treatment, the content of palmitic acid significantly decreased, and the two genes (g4988 and g11881) involved in the cutin, suberin, and wax biosynthesis pathway were significantly upregulated. In previous research of *Arabidopsis thaliana*, the g4988 gene was found to encode a cytochrome P450-dependent fatty acid ω-hydroxylase, which can catalyze the ω-hydroxylation of saturated and unsaturated fatty acids ranging from C12 to C18 [55]. The g11881 gene has been shown to encode a cytochrome P450 monooxygenase that plays a critical role in the synthesis of very long-chain ω-hydroxyacids and α, ω-dicarboxylic acids, which are essential components of suberin, a lipid polymer found in various plant tissues [56]. In the fatty acid biosynthesis pathway, the expression levels of g31191 and g60956 decreased, while the expression level of g49811 increased. The genes g31191 and g60956 encode proteins in *Saccharomyces cerevisiae* associated with β-ketoacyl synthase, an enzyme class typically involved in fatty acid synthesis [57]. On the other hand, g49811 encodes acyl-CoA synthetase, which catalyzes the reaction that combines long-chain fatty acids with coenzyme A to form acyl-CoA [58]. After treatment at 15 °C, the content of palmitic acid significantly increased, and the expression level of g19673 in the cutin, suberin, and wax biosynthesis pathway was significantly downregulated. This gene, belonging to the cytochrome P450 family, encodes a fatty acid ω-hydroxylase, which plays a role in the ω-hydroxylation of fatty acids [59]. After treatment at 25 °C, the content of palmitic acid decreased, and g59542 in the fatty acid biosynthesis pathway functioned similarly to g31191 and g60956.

In the linoleic acid synthesis pathway, the content of linoleic acid significantly decreased after the 25% shading treatment, and the genes g26575, g24787, and g23517 were significantly upregulated. The g26575 gene mainly catalyzes the hydrolysis of membrane glycerophospholipids in *A*. *thaliana* and *Oryza sativa*, releasing free fatty acids and lysophospholipids. These products are integral to cell signaling pathways, regulating plant growth, development, and responses to both abiotic and biotic stresses [60]. The g24787 gene encodes triacylglycerol lipase 4, an enzyme involved in the breakdown of stored fats [61]. The g23517 gene encodes a lipoxygenase (LOX) in *A*. *thaliana*, which is involved in the oxidation of fatty acids to produce fatty acid hydroperoxides [62]. After treatment at 25 °C, the content of linoleic acid decreased, the expression levels of genes g57649 and g58562 significantly increased, while the expression level of g4314 significantly decreased. The genes g57649 and g58562 encode the same enzyme as g24787. The g4314 gene, part of the lipoxygenase gene family in *Solanum tuberosum*, encodes an enzyme that plays a key role in fatty acid metabolism, particularly in leaves [63].

## 5. Conclusions

Our study indicated that two major components of sweet potato, palmitic acid and linoleic acid, are the main allelochemicals that exhibit strong allelopathic effects on seed germination, plant growth, antioxidant enzyme activity, and chlorophyll content of the two weed species we studied, *D. sanguinalis* and *B. pilosa*. The content of these two targeted metabolites was affected by different environmental conditions and was significantly increased under low temperature (15 °C). There are five metabolic pathways involved in targeted metabolites of fatty acids identified under different environmental conditions, including fatty acid biosynthesis, fatty acid elongation, fatty acid degradation, biosynthesis of cutin, suberine, and wax, and the linoleic acid metabolism pathway. The synthesis of palmitic acid is significantly enriched in the biosynthesis pathways of fatty acids, cutin, suberine, and wax, and the synthesis of linoleic acid is significantly enriched in the linoleic acid metabolism pathway. A total of 13 key expressed genes are located in the biosynthesis pathways of cutin, suberine, and wax, fatty acids and linoleic acid under different environmental conditions. The release and allelopathic effects of palmitic acid and linoleic acid may be regulated by multiple genes through the expression of related enzymes.

## Figures and Tables

**Table 1:** Effects of two components on the seed germination and growth of *Digitaria sanguinalis*.

Component	Concentration/(mg·mL^−1^)	Response Index			
		Germination	Root Length	Shoot Length	Biomass
Palmitic acid	0.125	−0.15 ± 0.02 a	−0.32 ± 0.04 a	−0.31 ± 0.02 a	−0.41 ± 0.04 a
	0.25	−0.17 ± 0.01 a	−0.37 ± 0.03 a	−0.42 ± 0.03 b	−0.57 ± 0.03 b
	0.50	−0.28 ± 0.03 b	−0.43 ± 0.04 a	−0.44 ± 0.04 b	−0.57 ± 0.05 b
	1.00	−0.40 ± 0.06 c	−0.62 ± 0.07 b	−0.76 ± 0.05 c	−0.86 ± 0.05 c
	2.00	−1.00 ± 0.00 d	−1.00 ± 0.00 c	−1.00 ± 0.00 d	−1.00 ± 0.00 d
Linoleic acid	0.125	−0.19 ± 0.04 a	−0.26 ± 0.03 a	−0.33 ± 0.03 a	−0.47 ± 0.03 a
	0.25	−0.51 ± 0.06 b	−0.48 ± 0.05 b	−0.46 ± 0.03 b	−0.75 ± 0.04 b
	0.50	−0.54 ± 0.05 b	−0.56 ± 0.05 b	−0.57 ± 0.03 c	−0.81 ± 0.05 b
	1.00	−0.66 ± 0.04 c	−0.80 ± 0.02 c	−0.83 ± 0.05 d	−0.91 ± 0.03 c
	2.00	−1.00 ± 0.00 d	−1.00 ± 0.00 d	−1.00 ± 0.00 e	−1.00 ± 0.00 d

Data are expressed as mean ± standard deviation. Different letters within the same column signify significant differences at *p* < 0.05.

**Table 2:** Effects of two components on the seed germination and growth of *Bidens pilosa.*

Component	Concentration/(mg·mL^−1^)	Response Index			
		Germination	Root Length	Shoot Length	Biomass
Palmitic acid	0.125	−0.10 ± 0.02 a	−0.06 ± 0.03 a	−0.01 ± 0.01 a	−0.42 ± 0.03 a
	0.25	−0.18 ± 0.04 b	−0.49 ± 0.05 b	−0.31 ± 0.05 b	−0.52 ± 0.05 a
	0.50	−0.25 ± 0.04 b	−0.66 ± 0.04 c	−0.43 ± 0.05 c	−0.69 ± 0.05 b
	1.00	−0.35 ± 0.03 c	−0.75 ± 0.03 c	−0.62 ± 0.04 d	−0.75 ± 0.05 b
	2.00	−1.00 ± 0.00 d	−1.00 ± 0.00 d	−1.00 ± 0.00 e	−1.00 ± 0.00 c
Linoleic acid	0.125	−0.16 ± 0.03 a	−0.26 ± 0.03 a	−0.18 ± 0.02 a	−0.48 ± 0.03 a
	0.25	−0.26 ± 0.02 b	−0.51 ± 0.04 b	−0.41 ± 0.03 b	−0.59 ± 0.04 b
	0.50	−0.36 ± 0.04 c	−0.53 ± 0.05 b	−0.43 ± 0.04 c	−0.66 ± 0.03 bc
	1.00	−0.46 ± 0.03 d	−0.77 ± 0.05 c	−0.68 ± 0.03 d	−0.75 ± 0.04 c
	2.00	−1.00 ± 0.00 e	−1.00 ± 0.00 d	−1.00 ± 0.00 e	−1.00 ± 0.00 d

Data are expressed as mean ± standard deviation. Different letters within the same column signify significant differences at *p* < 0.05.

**Table 3:** Antioxidant enzyme and chlorophyll properties of *Digitaria sanguinalis* affected by two components.

Component	Concentration/(mg·mL^−1^)	Effects of Antioxidant Enzyme and Chlorophyll				
		SOD (U·g^−1^)	CAT (U·g^−1^)	POD (U·g^−1^)	Chlorophyll a (mg·g^−1^)	Chlorophyll b (mg·g^−1^)
Palmitic acid	0.00	93.17 ± 2.26 a	82.06 ± 1.04 d	357.12 ± 3.03 a	1.51 ± 0.05 a	0.61 ± 0.02 a
	0.125	90.26 ± 1.95 a	88.29 ± 1.07 c	305.22 ± 1.52 b	1.25 ± 0.03 b	0.51 ± 0.02 b
	0.25	80.92 ± 1.02 b	90.54 ± 1.29 c	287.51 ± 1.08 c	1.14 ± 0.03 c	0.42 ± 0.01 c
	0.50	72.45 ± 1.04 c	121.08 ± 1.95 b	256.14 ± 1.02 d	0.74 ± 0.03 d	0.26 ± 0.01 d
	1.00	51.36 ± 1.02 d	156.22 ± 2.29 a	185.15 ± 1.38 e	0.65 ± 0.02 e	0.22 ± 0.01 e
Linoleic acid	0.00	93.17 ± 2.26 a	82.06 ± 1.74 e	357.12 ± 3.03 a	1.51 ± 0.05 a	0.61 ± 0.02 a
	0.125	89.33 ± 1.06 b	92.11 ± 1.09 d	289.56 ± 1.58 b	1.21 ± 0.03 a	0.46 ± 0.01 b
	0.25	78.28 ± 1.02 c	95.67 ± 1.09 c	284.39 ± 1.54 c	1.08 ± 0.03 b	0.38 ± 0.01 c
	0.50	65.95 ± 1.04 d	128.33 ± 2.04 b	207.59 ± 1.06 d	0.65 ± 0.02 c	0.24 ± 0.01 d
	1.00	50.61 ± 0.87 e	175.36 ± 2.65 a	182.74 ± 1.04 e	0.64 ± 0.02 c	0.22 ± 0.01 d

SOD = superoxide dismutase, CAT = catalase, POD = peroxidase. The different letters show significant differences at *p* < 0.05.

**Table 4:** Antioxidant enzyme and chlorophyll properties of *Bidens pilosa* affected by two components.

Component	Concentration/(mg·mL^−1^)	Effects of Antioxidant Enzyme and Chlorophyll				
		SOD (U·g^−1^)	CAT (U·g^−1^)	POD (U·g^−1^)	Chlorophyll a (mg·g^−1^)	Chlorophyll b (mg·g^−1^)
Palmitic acid	0.00	68.35 ± 1.02 e	205.18 ± 1.61 e	122.36 ± 1.16 e	0.56 ± 0.02 a	0.38 ± 0.01 a
	0.125	136.91 ± 2.06 d	226.05 ± 1.87 d	138.09 ± 1.51 d	0.53 ± 0.01 a	0.32 ± 0.01 b
	0.25	192.41 ± 2.02 c	301.06 ± 2.25 c	185.35 ± 1.68 c	0.49 ± 0.01 b	0.26 ± 0.01 c
	0.50	216.39 ± 2.24 a	356.15 ± 3.05 b	206.95 ± 1.55 b	0.43 ± 0.01 c	0.22 ± 0.01 d
	1.00	208.25 ± 1.98 b	402.28 ± 3.29 a	258.15 ± 1.61 a	0.42 ± 0.01 c	0.18 ± 0.01 e
Linoleic acid	0.00	68.35 ± 1.02 d	205.18 ± 1.61 e	122.36 ± 1.16 e	0.56 ± 0.02 a	0.38 ± 0.01 a
	0.125	142.42 ± 2.35 c	231.95 ± 1.91 d	184.06 ± 1.71 d	0.52 ± 0.01 b	0.29 ± 0.01 b
	0.25	235.17 ± 2.26 b	326.05 ± 1.89 c	227.45 ± 1.74 c	0.47 ± 0.01 c	0.21 ± 0.01 c
	0.50	241.25 ± 2.19 a	368.29 ± 2.96 b	265.52 ± 1.76 b	0.42 ± 0.01 d	0.16 ± 0.01 d
	1.00	241.09 ± 2.45 a	420.25 ± 3.36 a	284.76 ± 1.84 a	0.40 ± 0.01 d	0.15 ± 0.01 d

SOD = superoxide dismutase, CAT = catalase, POD = peroxidase. The different letters show significant differences at *p* < 0.05.

**Table 5:** The changes in the two targeted metabolites in Ningshu 25 under different environmental conditions.

Treatment	Percentage (%)	
	Palmitic Acid	Linoleic Acid
CK	6.52 ± 1.35 b	3.82 ± 0.51 a
25% Shading rate	4.81 ± 1.06 b	1.05 ± 0.22 b
15 °C	13.47 ± 1.22 a	4.09 ± 1.37 a
25 °C	3.62 ± 1.72 b	1.81 ± 0.58 b

Data are expressed as mean ± standard deviation. Different letters within the same column signify significant differences at *p* < 0.05.

**Table 6:** Differentially expressed genes in the biosynthesis pathways of cutin, suberine, and wax in Ningshu 25 under different environmental conditions.

Group	Gene ID	Log2FoldChange
25% Shading vs. CK	g4988	3.97457156716662
	g11881	3.35758208832697
15 °C vs. CK	g19673	−6.39521489126829
25 °C vs. CK	g4988	2.48950613189193

**Table 7:** Differential expressed genes in fatty acid biosynthesis pathways in Ningshu 25 under different environmental conditions.

Group	Gene ID	Log2FoldChange
25% Shading vs. CK	g31191	−1.55503605149456
	g60956	−2.67204997389706
	g49811	2.48878248318988
15 °C vs. CK	g60956	−3.31943014032762
25 °C vs. CK	g31191	−2.0097410236177
	g59542	−1.82598392163936
	g60956	−2.60482260069511

**Table 8:** Differential expressed genes in linoleic acid biosynthesis pathways in Ningshu 25 under different environmental conditions.

Group	Gene ID	Log2FoldChange
Shading vs. CK	g26575	2.35689972490559
	g24787	2.41051250392385
	g23517	2.2751367349408
15 °C vs. CK	g23517	−3.15358283138729
25 °C vs. CK	g57649	1.31114241352244
	g58562	1.6777062492831
	g4314	−1.41063225454408

## Data Availability

The raw sequence data reported in this paper have been deposited in the Genome Sequence Archive (Genomics, Proteomics & Bioinformatics 2021) in National Genomics Data Center (Nucleic Acids Res 2022), China National Center for Bioinformation/Beijing Institute of Genomics, Chinese Academy of Sciences (GSA: CRA017751) that are publicly accessible at https://ngdc.cncb.ac.cn/gsa (accessed on 31 December 2023).
